# Cardiovascular and renal outcomes of dual combination therapies with glucagon-like peptide-1 receptor agonists and sodium-glucose transport protein 2 inhibitors: a systematic review and meta-analysis

**DOI:** 10.1186/s12933-025-02900-8

**Published:** 2025-09-30

**Authors:** Arveen Shokravi, Jayant Seth, G. B. John Mancini

**Affiliations:** 1 Department of Medicine, University of British Columbia, Vancouver, BC V6T 1Z4 Canada; 2 Centre for Cardiovascular Innovation, Dilwari Cardiovascular Institute, Division of Cardiology, University of British Columbia, Vancouver, BC V5Z 1M9 Canada

**Keywords:** GLP-1 receptor agonist, SGLT2 inhibitor, Finerenone, Combination therapy, Cardiovascular outcomes, Renal outcomes, Type 2 diabetes, Chronic kidney disease, Meta-analysis, Cardiometabolic risk

## Abstract

**Background:**

Combination therapy with glucagon-like peptide-1 receptor agonists (GLP-1RA), sodium-glucose co-transporter 2 inhibitors (SGLT2i), and/or finerenone offers a strategy to reduce the risk of adverse cardiovascular and renal outcomes. This study aimed to quantify the cardiorenal benefits of combination regimens with GLP-1RA, SGLT2i, and/or finerenone versus corresponding monotherapies.

**Methods:**

MEDLINE and Embase were systematically searched, yielding four post hoc analyses of randomized controlled trials (RCTs) and ten observational studies that met prespecified inclusion criteria. Among RCTs, a random-effects meta-regression was performed to assess whether the effect of GLP-1RAs on cardiorenal outcomes differed based on baseline SGLT2i use. Additionally, for observational studies, random-effects meta-analyses were performed to estimate the effect of combination therapy versus monotherapy on the risk of cardiorenal outcomes.

**Results:**

Across RCTs, p for interaction was > 0.05 for major adverse cardiac events (MACE) (*p* = 0.730), cardiovascular (CV) mortality (*p* = 0.889), non-fatal myocardial infarction (MI) (*p* = 0.237), non-fatal stroke (*p* = 0.696), all-cause mortality (*p* = 0.682), heart failure (HF) hospitalization (*p* = 0.257), and renal composite outcome (*p* = 0.890), supporting that GLP-1RAs result in a consistent reduction in outcomes irrespective of baseline SGLT2i use. In observational trials, compared to SGLT2i monotherapy, GLP-1RA and SGLT2i combination therapy significantly reduced MACE (HR 0.59, 95% CI 0.47–0.75), MI (HR 0.73, 95% CI 0.61–0.88), stroke (HR 0.72, 95% CI 0.53–0.97), all-cause mortality (HR 0.57, 95% CI 0.48–0.67), and HF hospitalization/events (HR 0.71, 95% CI 0.59–0.86). Compared to GLP-1RA monotherapy, SGLT2i and GLP-1RA combination therapy significantly reduced CV mortality (HR 0.35, 95% CI 0.15–0.81), MI (HR 0.93, 95% CI 0.88–0.97), stroke (HR 0.92, 95% CI 0.88–0.96), all-cause mortality (HR 0.59, 95% 0.49–0.70), HF hospitalization/events (HR 0.84, 95% CI 0.81–0.88), and serious renal events (HR 0.43, 95% CI 0.23–0.80). Compared to either SGLT2i or finerenone monotherapy, SGLT2i and finerenone combination therapy significantly reduced all-cause mortality and major adverse kidney events.

**Conclusion:**

Combination therapy with GLP-1RA, SGLT2i, or finerenone confers cardiorenal protection beyond monotherapy in T2D, as supported by concordant evidence from RCTs and large real-world cohorts. These findings support broader clinical adoption of dual-agent strategies but also underscore the need for dedicated prospective trials powered to assess hard clinical outcomes with dual-agent strategies.

**Supplementary Information:**

The online version contains supplementary material available at 10.1186/s12933-025-02900-8.

## Introduction

Cardiometabolic disorders remain a leading cause of premature morbidity and mortality worldwide [1, 2]. The interconnected nature of cardiovascular, kidney, and metabolic diseases necessitates a shift towards comprehensive risk-modifying strategies. Glucagon-like peptide-1 receptor agonists (GLP-1RA) and sodium-glucose cotransporter 2 inhibitors (SGLT2i) have each demonstrated cardiovascular and renal benefits across high-risk populations, including those with type 2 diabetes (T2D), atherosclerotic cardiovascular disease (ASCVD), heart failure (HF), and chronic kidney disease (CKD). Additionally, finerenone, a non-steroidal mineralocorticoid receptor antagonist (MRA), has shown benefit in patients with diabetic kidney disease (DKD) and heart failure with preserved ejection fraction (HFpEF) [3–5]. The distinct mechanisms of actions and expanding indications of these drug classes have led to increasing clinical interest and provide compelling rationale for combination therapy.

While contemporary guidelines support the use of SGLT2i and GLP-1RA agents in patients with overlapping cardiometabolic risk profiles [6–8], uptake of dual therapy remains limited in clinical practice [9], due in part to uncertainty regarding its incremental benefit over monotherapy. Some societies have discussed adding non-steroidal MRAs to SGLT2i in patients with T2D and CKD [10]; however, given its relatively recent approval, obstacles remain to ensuring all indicated patients receive treatment [11]. Moreover, randomized controlled trials (RCTs) evaluating whether concurrent use confers additive or synergistic benefits on hard clinical outcomes beyond either agent alone remain sparse. Although subgroup and post hoc analyses suggest a favorable signal, few studies have systematically examined the clinical outcomes associated with combination therapy. Additionally, existing observational studies examining hard clinical outcomes are limited and heterogeneous in terms of population, follow-up duration, and endpoints, complicating clinical interpretation. Nonetheless, several new studies have been published in the past year that may help address these gaps.

Accordingly, this review aims to comprehensively evaluate the cardiovascular and renal outcomes of dual combination therapies involving GLP-1RA, SGLT2i, or finerenone, compared to monotherapy. This analysis focuses on RCTs of GLP-1RAs that report baseline presence or absence of SGLT2i. The analysis is further supplemented by observational studies assessing dual versus monotherapy in routine clinical settings. By synthesizing evidence across trial designs and populations, this study aims to clarify the clinical impact of combination therapy and inform future treatment strategies.

## Methods

This review was conducted in accordance with the Preferred Reporting Items for Systematic Reviews and Meta-Analysis (PRISMA) [12].

A comprehensive search was conducted across MEDLINE and Embase from date of inception to June 1st 2025. The full search strategy is included in the supplementary appendix. RCTs and observational trials were included in this review. RCTs of interest were limited to GLP-1RA trials with data on baseline SGLT2i status, including any data from post hoc analyses. SGLT2i RCTs with data on baseline GLP-1RA status were not included in this review given the recent comprehensive SMART-C trial published in 2024 [13]. Similarly, trial-level data from the landmark finerenone RCTs—FIDELIO, FIGARO, and FINE-ARTS—examining dual therapy with SGLT2i or GLP-1RA alongside finerenone have been recently meta-analyzed [14–16]. Given the thoroughness of these analyses and lack of new RCT evidence, we elected to limit our synthesis of RCTs to GLP-1RA trials. Additionally, observational trials examining the effects of dual therapy with GLP-1RA, SGLT2i, and/or finerenone, compared to monotherapy, were included in this review. Inclusion criteria include one or more clinical outcomes of interest, including major adverse cardiac events (MACE), all-cause mortality, myocardial infarction (MI), stroke, cardiovascular (CV) mortality, heart failure (HF) outcomes (including HF hospitalization or HF events), and renal outcomes including renal composite outcomes, serious renal events, or major adverse kidney events (MAKE). When multiple hazard ratios (HRs) were reported for a given outcome, multivariable-adjusted HRs were prioritized. The populations of interest included adults with T2D, CKD, or HF; however, the final study population primarily consisted of patients with T2D or studies in which the majority of participants (> 85%) had T2D. Studies that focused solely on soft or surrogate endpoints or examined pediatric populations were excluded from this review.

Studies were uploaded to Covidence, and two authors independently screened abstracts, completed full text review, and conducted data extraction. Any conflicts during this process were resolved via consensus. The Cochrane Risk of Bias 2.0 (RoB 2.0) tool [17] was used to assess the methodological quality of included RCTs and the Newcastle Ottawa Scale was used to assess observational studies [18].

Statistical analysis and forest plot generation was completed using the RStudio software (version 4.3.2) and meta and metafor packages [19, 20]. Meta-analyses were conducted using random effects models with restricted maximum likelihood estimation. For analysis of RCT trials, heterogeneity in treatment effects by baseline SGLT2i status was assessed using meta-regression with the metafor package [19]. Forest plots presented hazard ratios (HR) with corresponding 95% confidence intervals (CI), with statistical significance defined as a *p*-value < 0.05. Heterogeneity was assessed using the chi-squared (Cochran’s Q) test (α = 0.10), and its magnitude was quantified using the I^2^ statistic. Log-transformed HRs and corresponding standard errors from randomized controlled trials were modeled using a random-effects meta-regression with restricted maximum likelihood estimation and Hartung-Knapp adjustment.

Additionally, for the observational study data, we conducted subgroup analyses to explore potential sources of heterogeneity. For subgroup analysis, studies were stratified by baseline risk into two groups: (1) lower-risk T2D populations and (2) populations with T2D and established cardiovascular disease (e.g., MI, ACS, or ASCVD).

## Results

### Study selection process

A total of 3161 studies were retrieved from MEDLINE and EMBASE, with an additional one identified through gray literature sources. After removal of duplicates, 2872 unique records remained. Title and abstract screening yielded 29 articles for full-text assessment. Of these, 14 met the eligibility criteria and were included for data extraction. This final set comprised four post hoc analyses [21–24] of four RCTs [25–28], and ten observational studies [29–38]. The study selection process is illustrated in the PRISMA diagram (Supplementary Figure S1).

### Baseline study information

Baseline information for the included RCTs is presented in Supplementary Table S1, and for the included observational studies in Supplementary Table S2 and S3. All RCT data included T2D populations, with AMPLITUDE-O, Harmony Outcomes, and SOUL examining patients with concurrent CVD or ASCVD history, and FLOW enrolling patients with concurrent CKD. All of the included observational trials represented diabetic populations or populations with high (> 85%) incidence of T2D. Many of the included observational trials included patients with additional comorbidities such as history of acute coronary syndrome (ACS), MI, ASCVD, heart failure with reduced ejection fraction (HFrEF), or HFpEF. Supplementary Table S4 summarizes the populations of the included observational trials.

### Risk of bias

The Cochrane RoB 2.0 tool was used to assess the included RCTs. All studies were deemed to be low risk overall, and low risk for all domains including randomization process, intervention deviation, missing outcome data, measurement of outcomes, and reporting of the outcome. Newcastle Ottawa Scale was used to assess the included observational trials. Most studies received a score of 8 out of 9, with deductions typically due to inadequate follow-up or failure to demonstrate that the outcomes of interest had never occured prior to the start of the study. The full risk of bias tables can be found in the supplementary appendix tables S5 and S6.

### Randomized control trial data

#### Major adverse cardiovascular events

**Fig. 1:**
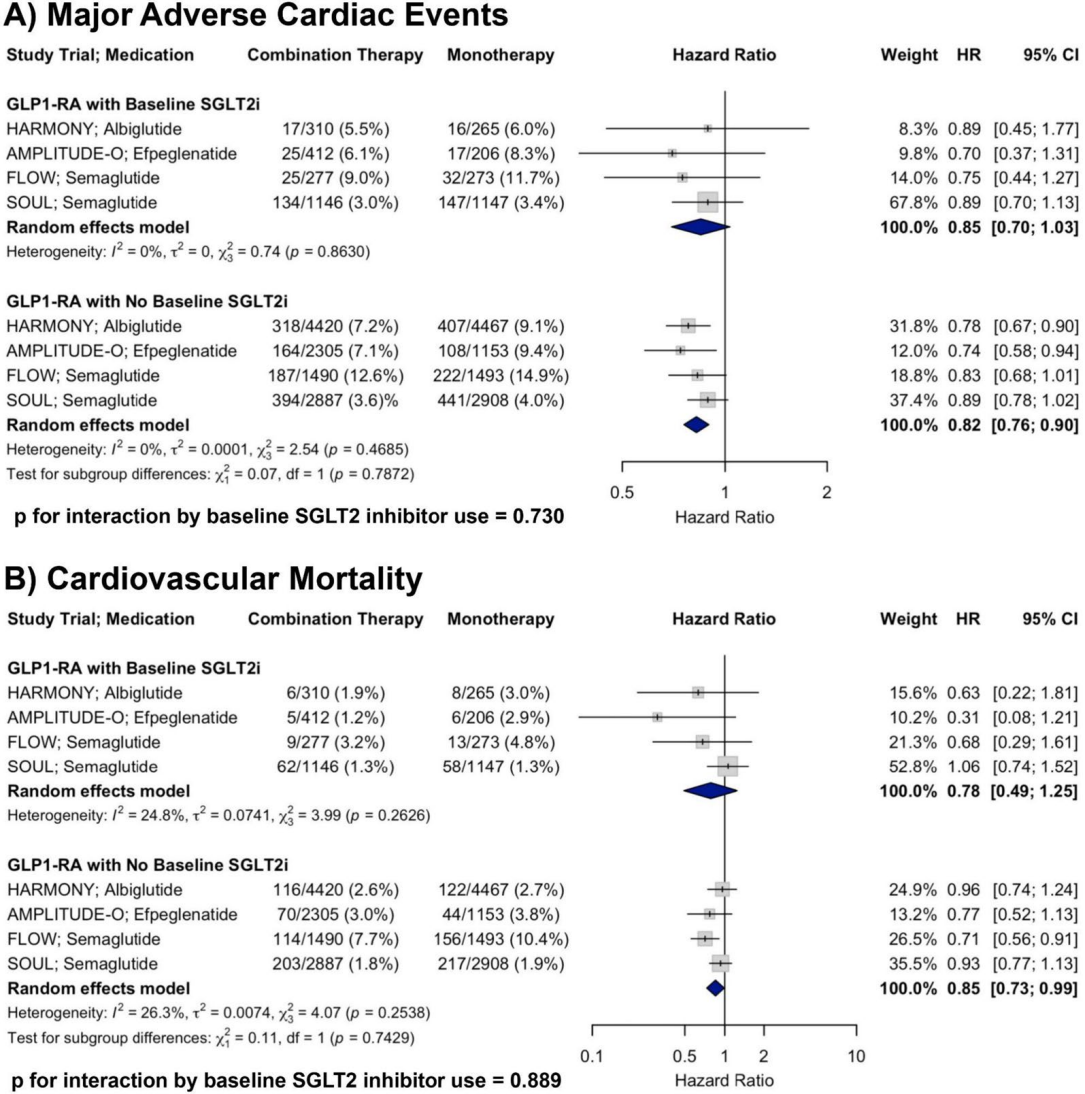
Forest plots showing the effects of GLP-1RA therapy on **A** MACE and **B** CV mortality across RCTs, stratified by baseline SGLT2i use, with *p*-values for interaction shown

A total of four RCTs were included in the analysis of MACE. In patients with baseline SGLT2i use receiving GLP-1RA therapy, the pooled HR was 0.85 (95% CI: 0.70–1.03) compared to placebo. In patients receiving GLP-1RA therapy without baseline SGLT2i, the pooled HR was 0.82 (95% CI: 0.76–0.90). There was no evidence of heterogeneity within either subgroup (I^2^ = 0% for both). Meta-regression showed no significant interaction by baseline SGLT2i use (p for interaction = 0.730). Although only one subgroup reached statistical significance, the non-significant interaction *p*-value (*p* = 0.730) indicates no strong evidence of a differential treatment effect of GLP-1RA based on baseline SGLT2i use (Fig. 1, Panel A).

#### Cardiovascular mortality

A total of four RCTs were included in the analysis of CV mortality. In patients with baseline SGLT2i use receiving GLP-1RA therapy, the pooled HR was 0.78 (95% CI: 0.49–1.25) compared to placebo. In patients receiving GLP-1RA therapy without baseline SGLT2i, the pooled HR was 0.85 (95% CI: 0.73–0.99). There was minimal to moderate heterogeneity within subgroups (I^2^ = 24.8% and 26.3%, respectively). There was no significant interaction by baseline SGLT2i use (*p* for interaction = 0.889), supporting a consistent reduction in CV mortality with GLP-1RA therapy irrespective of concurrent SGLT2i treatment (Fig. 1, Panel B).

#### Non-fatal myocardial infarction

**Fig. 2:**
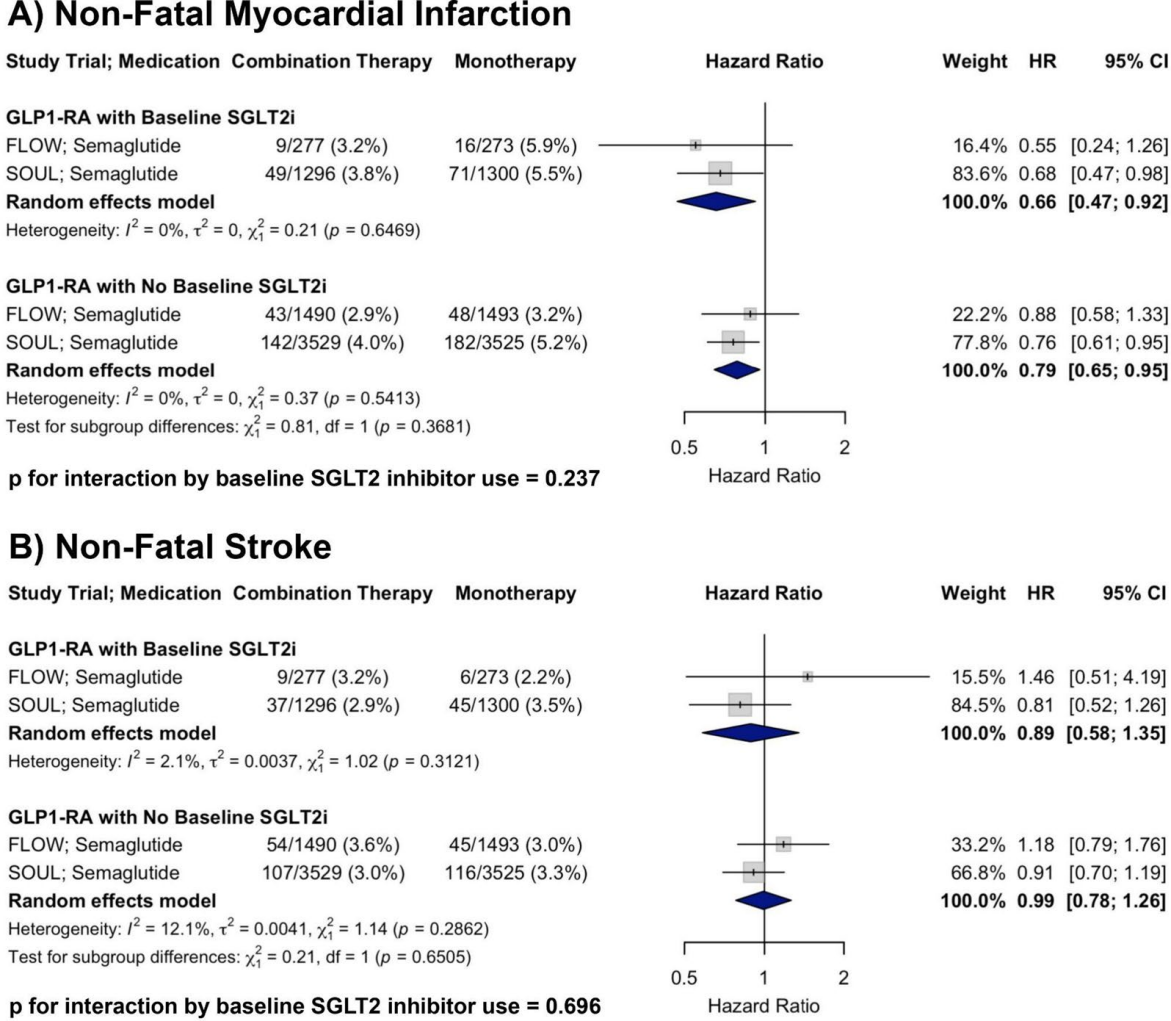
Forest plots showing the effects of GLP-1RA therapy on **A** non-fatal MI and **B** non-fatal stroke across RCTs, stratified by baseline SGLT2i use, with *p*-values for interaction shown

A total of two RCTs were included in the analysis of non-fatal MI. In patients with baseline SGLT2i use receiving GLP-1RA therapy, the pooled HR was 0.66 (95% CI: 0.47–0.92) compared to placebo. In patients receiving GLP-1RA therapy without baseline SGLT2i, the pooled HR was 0.79 (95% CI: 0.65–0.95). There was no evidence of heterogeneity within either subgroup (I^2^ = 0% for both). There was no significant interaction by baseline SGLT2i use (p for interaction = 0.237), supporting a consistent reduction in non-fatal MI with GLP-1RA therapy irrespective of concurrent SGLT2i treatment (Fig. 2, Panel A).

#### Non-fatal stroke

A total of two RCTs were included in the analysis of non-fatal stroke. In patients with baseline SGLT2i use receiving GLP-1RA therapy, the pooled HR was 0.89 (95% CI: 0.58–1.35) compared to placebo. In patients receiving GLP-1RA therapy without baseline SGLT2i, the pooled HR was 0.99 (95% CI: 0.78–1.26). There was minimal heterogeneity within subgroups (I^2^ = 2.1% and 12.1%, respectively). There was no significant interaction by baseline SGLT2i use (*p* for interaction = 0.696), suggesting a similar effect of GLP-1RA therapy on non-fatal stroke irrespective of baseline SGLT2i treatment (Fig. 2, Panel B).

#### All-cause mortality

**Fig. 3:**
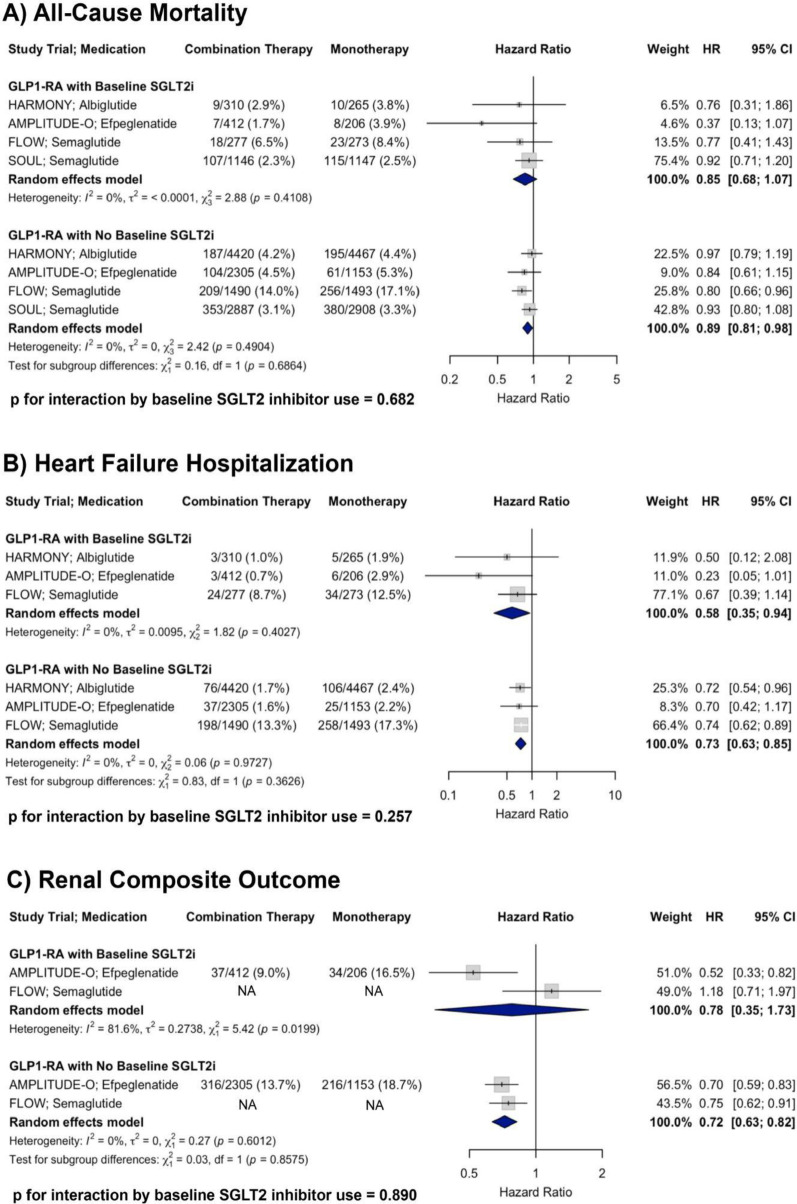
Forest plots showing the effects of GLP-1RA therapy on **A** all-cause mortality, **B** HF hospitalization, and **C** renal composite outcome across RCTs, stratified by baseline SGLT2i use, with *p*-values for interaction shown

A total of four RCTs were included in the analysis of all-cause mortality. In patients with baseline SGLT2i use receiving GLP-1RA therapy, the pooled HR was 0.85 (95% CI: 0.68–1.07) compared to placebo. In patients receiving GLP-1RA therapy without baseline SGLT2i, the pooled HR was 0.89 (95% CI: 0.81–0.98). There was no evidence of heterogeneity within either subgroup (I^2^ = 0% for both). There was no significant interaction by baseline SGLT2i use (p for interaction = 0.682), supporting a consistent reduction in all-cause mortality with GLP-1RA therapy irrespective of concurrent SGLT2i treatment (Fig. 3, Panel A).

#### Heart failure hospitalization

A total of three randomized controlled trials were included in the analysis of HF hospitalization. In patients with baseline SGLT2i use receiving GLP-1RA therapy, the pooled HR was 0.58 (95% CI: 0.35–0.94) compared to placebo. In patients receiving GLP-1RA therapy without baseline SGLT2i, the pooled HR was 0.73 (95% CI: 0.63–0.85). There was no evidence of heterogeneity within either subgroup (I^2^ = 0% for both). There was no significant interaction by baseline SGLT2i use (p for interaction = 0.257), supporting a consistent reduction in HF hospitalization with GLP-1RA therapy irrespective of concurrent SGLT2i treatment (Fig. 3, Panel B).

#### Renal composite outcome

A total of two RCTs were included in the analysis of the renal composite outcome. The AMPLITUDE-O renal composite definition included incident macroalbuminuria, a sustained decrease in estimated glomerular filtration rate (eGFR) by ≥ 40% for at least 30 days, initiation of renal replacement therapy, or a sustained eGFR < 15 mL/min/1.73 m^2^ for at ≥ 30 days. FLOW defined the renal outcome as a four-component composite consisting of the onset of a sustained ≥ 50% reduction in eGFR from baseline, kidney failure (initiation of chronic dialysis, kidney transplantation, or a sustained reduction in eGFR < 15 mL/min/1.73 m^2^ for at ≥ 28 days), or death due to kidney causes. In patients with baseline SGLT2i use receiving GLP-1RA therapy, the pooled HR was 0.78 (95% CI: 0.35–1.73). In patients with baseline SGLT2i use receiving GLP-1RA therapy, the pooled HR was 0.72 (95% CI: 0.63–0.82). There was substantial heterogeneity in the subgroup with SGLT2i use (I^2^ = 81.6%), but not in those without (I^2^ = 0%). No significant interaction by SGLT2i use was observed (*p* for interaction = 0.890), indicating that GLP-1RA therapy was associated with a consistent outcome regardless of concomitant SGLT2i treatment (Fig. 3, Panel C).

### Observational trial data (SGLT2i and GLP-1RA combination therapy)

#### Major adverse cardiovascular events

**Fig. 4:**
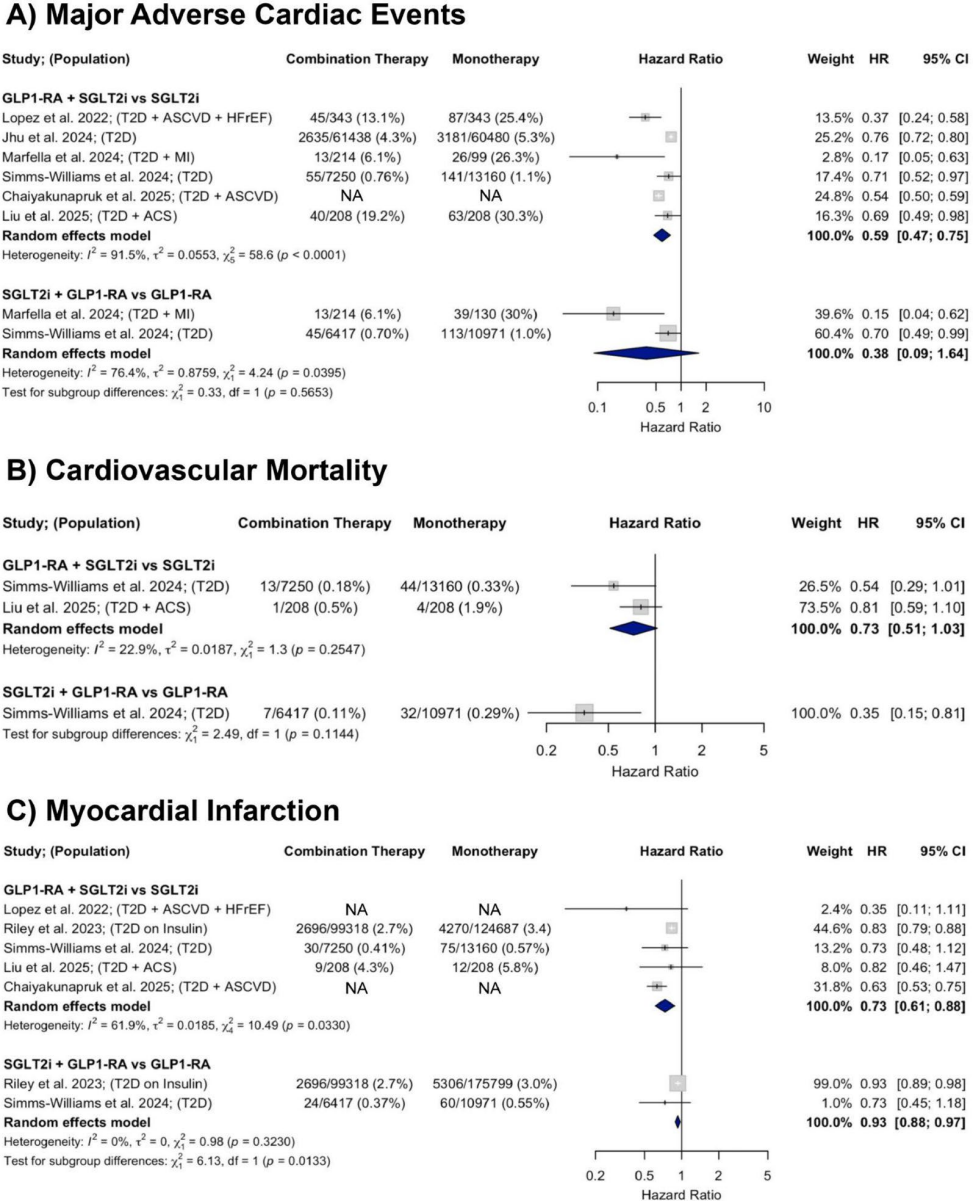
Forest plots showing the effects of GLP-1RA and SGLT2i combination therapy versus monotherapy on **A** MACE, **B** CV mortality, and **C** MI across observational studies

A total of six observational studies were included in the analysis of MACE. In studies comparing GLP-1RA and SGLT2i combination therapy to SGLT2i monotherapy, there was a significant 41% decrease in MACE (HR = 0.59; 95% CI 0.47–0.75). In those comparing combination therapy to GLP-1RA monotherapy, there was a non-significant downward trend in MACE incidence with a pooled HR of 0.38 (95% CI: 0.09–1.64). Heterogeneity was substantial across both comparisons (I^2^ = 91.5% and 76.4%, respectively) (Fig. 4, Panel A).

#### Cardiovascular mortality

A total of two observational studies were included in the analysis of CV mortality. In studies comparing GLP-1RA and SGLT2i combination therapy to SGLT2i monotherapy, there was a non-significant downward trend in CV mortality (HR = 0.73; 95% CI 0.51–1.03) with low heterogeneity (I^2^ = 22.9%). One study compared GLP-1RA and SGLT2i combination therapy to GLP-1RA monotherapy and found a significant 65% decrease in CV mortality risk (HR = 0.35; 95% CI 0.15–0.80) (Fig. 4, Panel B).

#### Myocardial infarction

A total of five observational studies were included in the analysis of myocardial infarction. In studies comparing GLP-1RA and SGLT2i combination therapy to SGLT2i monotherapy, there was a 27% decrease in MI incidence (HR = 0.73; 95% CI 0.61–0.88) with substantial heterogeneity (I^2^ = 61.9%). In those comparing GLP-1RA and SGLT2i combination therapy to GLP-1RA monotherapy, there was a 7% decrease in MI risk (HR = 0.93; 95% CI 0.88–0.97) with no heterogeneity (I^2^ = 0%) (Fig. 4, Panel C).

#### Stroke

**Fig. 5:**
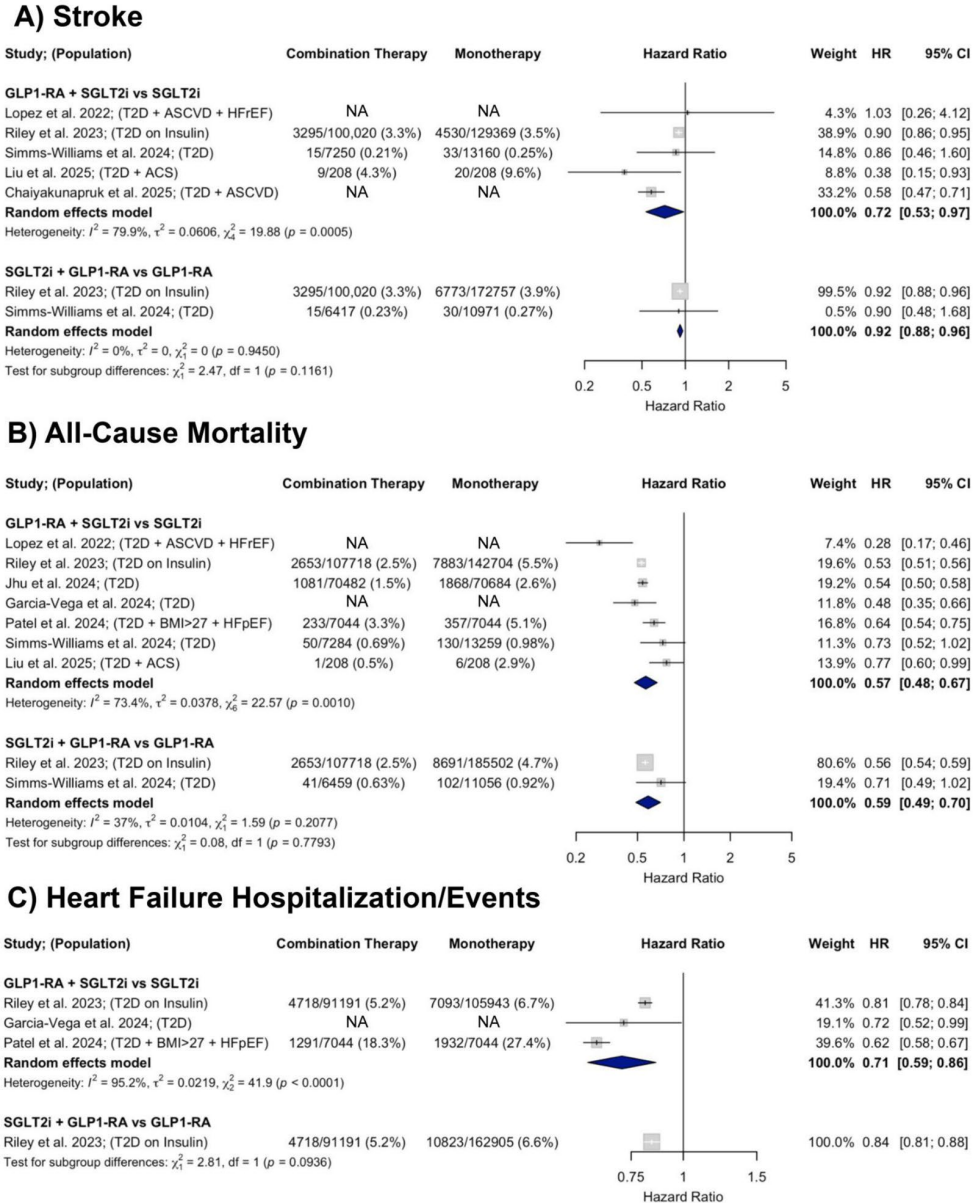
Forest plots showing the effects of GLP-1RA and SGLT2i combination therapy versus monotherapy on **A** stroke, **B** all-cause mortality, and **C** HF hospitalization/events across observational studies

A total of five observational studies were included in the analysis of stroke. In studies comparing GLP-1RA and SGLT2i combination therapy to SGLT2i monotherapy, there was a 28% decrease in stroke risk (HR = 0.72; 95% CI 0.53–0.97) with considerable heterogeneity (I^2^ = 79.9%). In those comparing GLP-1RA and SGLT2i combination therapy to GLP-1RA monotherapy, there was a 8% decrease in stroke risk (HR = 0.92; 95% CI 0.88–0.96) with no heterogeneity (I^2^ = 0%) (Fig. 5, Panel A).

#### All-cause mortality

A total of seven observational studies were included in the analysis of all-cause mortality. In studies comparing GLP-1RA and SGLT2i combination therapy to SGLT2i monotherapy, there was a significant 43% decrease in all-cause mortality (HR = 0.57; 95% CI 0.48–0.67). In those comparing GLP-1RA and SGLT2i combination therapy to GLP-1RA monotherapy, there was a 41% decrease in all-cause mortality (HR = 0.59; 95% CI 0.49–0.70). Heterogeneity was moderate to substantial across comparisons (I^2^ = 73.4% and 37%, respectively) (Fig. 5, Panel B).

#### Heart failure hospitalization/events

A total of three observational studies were included in the analysis of HF hospitalization or events. In studies comparing GLP-1RA and SGLT2i combination therapy to SGLT2i monotherapy, there was a significant 29% decrease in time to first HF hospitalization/event (HR = 0.71; 95% CI 0.59–0.86) with substantial heterogeneity (I^2^ = 95.2%). One study compared GLP-1RA and SGLT2i combination therapy to GLP-1RA monotherapy and found a 16% decrease in time to first HF hospitalization (HR = 0.84; 95% CI 0.80–0.87) (Fig. 5, Panel C).

#### Serious renal events

One study compared the effect of GLP-1RA and SGLT2i combination therapy to monotherapy on serious renal events, which included incidence of acute kidney injury, CKD, kidney failure, chronic hypertensive renal disease, and renal complications of diabetes. In patients with T2D, combination therapy was associated with a HR of 0.43 (95% CI 0.23–0.80) when compared to GLP-1RA monotherapy, and a HR of 0.67 (95% CI 0.32–1.41) when compared to SGLT2i monotherapy.

#### Subgroup analysis

Given the heterogeneity across study populations, subgroup analyses were conducted. Two subpopulations were examined: (1) a lower-risk T2D population, defined as studies enrolling a broad T2D cohort or general population with high T2D incidence, without requiring additional comorbidities or high risk features (e.g. MI history or insulin use); and (2) a population with T2D and vascular or coronary disease, defined as studies including participants with comorbid ACS, MI, or ASCVD. Forest plots and HRs with 95% CIs are presented in the supplementary appendix (Supplemental Figures S2-S12).

In the first subgroup, only MACE and all-cause mortality had sufficient data for statistical analysis comparing GLP-1RA and SGLT2i combination therapy to SGLT2i monotherapy. MACE was significantly reduced by 24% (HR = 0.76; 95% CI 0.72–0.80), with no observed heterogeneity (I^2^ = 0%) (Supplementary Figure S2). All-cause mortality was also significantly reduced by 44% (HR = 0.56; 95% CI 0.48–0.65); heterogeneity was moderate (I^2^ = 45.2%) but lower than that observed in the overall population (Supplementary Figure S10).

In the T2D with vascular or coronary disease subgroup, MACE, MI, stroke, and all-cause mortality had sufficient data for analysis comparing GLP-1RA and SGLT2i combination therapy to SGLT2i monotherapy. MACE was significantly reduced by 50% (HR = 0.50; 95% CI 0.36–0.69), with moderate heterogeneity (I^2^ = 61.1%) that was lower than in the overall population (Supplementary Figure S3). MI and stroke were significantly reduced by 36% (HR = 0.64; 95% CI 0.54–0.75) and 43% (HR = 0.57; 95% CI 0.47–0.70), respectively, with no heterogeneity (I^2^ = 0% for both). In this subpopulation, all-cause mortality lost significance (HR = 0.48; 95% CI 0.18–1.28) and heterogeneity became considerable (I^2^ = 92%) (Supplementary Figure S11).

### Observational trial data (finerenone and SGLT2i combination therapy)

One study compared finerenone and SGLT2i combination therapy to using either medication alone [29]. This study examined MACE, all-cause mortality, and major adverse kidney events (MAKE) which was defined as stage 5 CKD, or need for dialysis therapy or renal transplantation. Patients were propensity score matched; all patients had CKD, and the majority had T2D, with prevalence ranging from 87.1 to 89.2% across treatment groups.

#### Finerenone and SGLT2i combination therapy vs. SGLT2i monotherapy

During a mean follow-up of 12.2 ± 9.7 months, combined finerenone and SGLT2i group had a 56% reduction in MAKE risk compared to SGLT2i monotherapy (HR = 0.44; 95% CI 0.22–0.89). All-cause mortality was 59% lower in the combination therapy group compared to SGLT2i monotherapy (HR = 0.41; 95% CI 0.17–0.96). There was no significant difference in MACE between treatment groups (HR = 0.66 95% CI 0.27–1.61).

#### Finerenone and SGLT2i combination therapy vs. finerenone monotherapy

During a mean follow-up of 13.0 ± 9.9 months, combined finerenone and SGLT2i group had a 80% reduction in MAKE risk compared to finerenone monotherapy (HR = 0.20; 95% CI 0.09–0.45). All-cause mortality was 69% lower in the combination therapy group compared to finerenone monotherapy (HR = 0.31, 95% CI 0.12–0.82). There was no significant difference in MACE between treatment groups (HR = 0.77, 95% CI 0.27–2.26).

## Summary of results

**Fig. 6:**
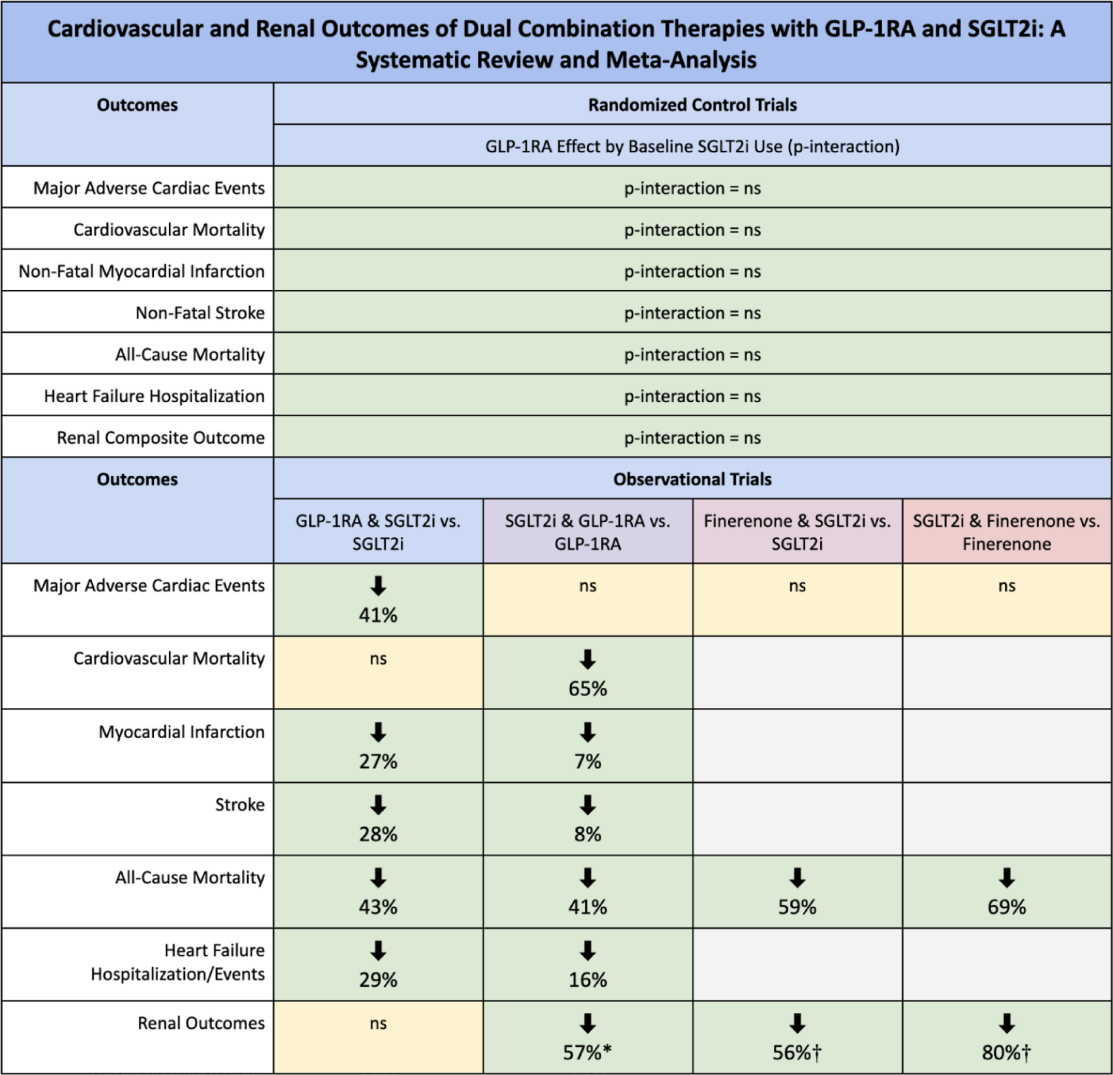
Summary of RCT and observational trial results. GLP-1RA = Glucagon-like peptide-1 receptor agonist; SGLT2i = Sodium-glucose transport protein 2 inhibitors; ns = Not significant; * Serious renal events: acute kidney injury, chronic kidney disease, kidney failure, chronic hypertensive renal disease, and renal complications of diabetes; † Major adverse kidney event: stage 5 CKD, or need for dialysis therapy or renal transplantation

Figure 6 provides a visual summary of results of the analyses stratified by RCTs and observational trials.

## Discussion

### Randomized trial findings

This meta-analysis of four RCTs evaluating GLP-1 receptor agonists in individuals with T2D demonstrates consistent effects on several cardiorenal outcomes, irrespective of baseline SGLT2i use. Our findings are concordant with prior work by Neuen et al., which included data from Harmony Outcomes, AMPLITUDE-O and FLOW [39]. Our study extends the evidence base by incorporating new data from the SOUL trial. Meta-regression analyses showed no significant interaction between baseline SGLT2i use and the treatment effect of GLP-1RA therapy across key endpoints, including MACE, CV mortality, non-fatal MI, non-fatal stroke, all-cause mortality, HF hospitalization, and renal composite outcome. This suggests that GLP-1RA therapies confer additive cardioprotective benefit regardless of concurrent SGLT2i therapy.

Although the included RCTs were not specifically designed to evaluate the efficacy of GLP-1RA and SGLT2i combination therapy, nonetheless, the consistent directionality of treatment effects across subgroups stratified by baseline SGLT2i use for several outcomes highlight the potential utility of dual-agent strategies. These findings should be interpreted in the context and limitations of post hoc analyses. Definitive conclusions regarding the incremental benefit of combination therapy on hard clinical endpoints will require prospective trials explicitly designed for this purpose. That said, several randomized trials have assessed surrogate endpoints. A prior meta-analysis of eight RCTs demonstrated that compared to monotherapy, combination therapy GLP-1RA and SGLT2i resulted in greater reductions in hemoglobin A1c, fasting plasma glucose, low-density lipoprotein cholesterol, and systolic blood pressure [40].

Analysis of SGLT2 inhibitor RCTs showed consistent reductions in MACE, HF hospitalization, and CKD progression regardless of baseline GLP-1RA use, as demonstrated by a recent SMART-C consortium analysis of over 73,000 individuals with T2D [13]. Similarly, finerenone RCTs were not included in our analysis, as previous studies have already conducted comprehensive analyses, and no new data relevant to our analysis have been published since [14–16]. Pooled analyses of the FIGARO and FIDELIO trials, which evaluated the efficacy of finerenone in DKD, found that the *p*-values for interaction were all > 0.05 for both cardiovascular and kidney composite outcomes, regardless of whether finerenone was used with or without GLP-1RA or SGLT2i [14, 15]. This suggests that finerenone’s efficacy is maintained regardless of baseline GLP-1RA or SGLT2i use. The recently published CONFIDENCE trial directly randomized patients with CKD and T2D to finerenone and empagliflozin combination therapy or monotherapy [41]. Although no hard clinical endpoints were assessed, combination therapy resulted in a 29% greater reduction in albumin-to-creatinine ratio compared to finerenone monotherapy, and a 32% greater reduction compared to empagliflozin monotherapy [41]. The observed reductions in albuminuria have prognostic significance, as prior meta-analytic data suggest a 30% reduction is associated with a 27% lower risk of kidney composite outcomes [42].

Finerenone also has evidence for efficacy in HFpEF. A post hoc analysis from the FINE-ARTS trial, which included patients with HFmrEF or HFpEF, found a *p*-value for interaction > 0.05 between patients with and without baseline SGLT2i use for the composite outcome of total worsening HF events and CV death [43], suggesting that the benefits of finerenone were consistent regardless of baseline SGLT2i therapy. The CONFIRMATION-HF trial is a RCT currently being conducted to evaluate the efficacy of combination therapy with finerenone and an SGLT2i in the broader HF population [44].

### Observational evidence

While the randomized trials in this analysis relied on meta-regression stratified by baseline SGLT2i use to assess potential treatment effect differences, the observational studies enabled more direct statistical comparisons between combination therapy and monotherapy. The real-world data consistently suggested that dual therapy is associated with greater reductions in various cardiorenal outcomes.

These findings provide complementary insights into the effectiveness of combination therapy in routine clinical settings, where treatment decisions are shaped by individual risk profiles and therapeutic goals. The observational studies capture a broader range of patients with T2D, including those with MI history or advanced disease on insulin. As such, they offer valuable external validity and may better reflect the potential population-level benefits of dual therapy implementation. However, substantial heterogeneity was observed across several outcomes, and interpretation must be tempered by the inherent limitations of observational study design [45]. Despite these limitations, the consistency of findings across diverse populations and clinical settings strengthens the case for combination therapy as a viable strategy in high-risk patients. When considered alongside the biological plausibility of synergistic mechanisms and supportive trial data, these observational results reinforce the need for a dedicated randomized trial powered to evaluate the incremental benefit of dual therapy on hard clinical outcomes.

Observational data examining hard endpoints for finerenone combination therapy were limited to one study that investigated finerenone and SGLT2i combination therapy [29]. It found significant reductions in MAKE and all-cause mortality compared to monotherapy, along with a trend toward decreased MACE [29].

Although both observational and RCT data suggest a benefit of SGLT2i and GLP-1RA combination therapy, a greater number of outcomes reached significance in the observational comparison of combination therapy versus SGLT2i monotherapy, compared to RCT subgroup analyses of GLP1-RA versus placebo in patients with baseline SGLT2i. This discrepancy may result from differences in study design. While the observational data compares treatment strategies in broader clinical populations, the RCT data reflect subgroup analyses not explicitly designed to evaluate combination therapy. Additionally, other factors such as differences in baseline risk, inclusion criteria, and follow-up periods may further explain the difference in effect size. The consistent direction of benefit across study designs, albeit with varying magnitudes and statistical significance, supports the efficacy of SGLT2i and GLP-1RA combination therapy; however, the extent of benefit requires further evidence from trials specifically designed to evaluate combination therapy.

### Clinical implications

Mechanistically, SGLT2i, GLP-1RA, and finerenone act through distinct pathways, supporting the rationale for combined therapy to enhance clinical outcomes. SGLT2i act primarily by inhibiting glucose and sodium reabsorption in the proximal tubule, leading to natriuresis, glucosuria, and enhanced tubuloglomerular feedback [46]. Additionally, SGLT2i have anti-inflammatory and antifibrotic properties, and improve myocardial and renal energy metabolism [47]. Similarly, GLP-1RAs reduce cardiorenal risk through a combination of direct and indirect mechanisms. Their primary mechanisms include improved glycemic control and weight loss, which reduces blood pressure and has favorable effects on lipid profiles, all of which contribute to lower cardiorenal risk [48]. GLP-1RAs also reduce renal events in part due to improved intrarenal hemodynamics [49], and limit CV events through direct antiatherosclerotic effects [50]. Finerenone works by selectively antagonizing mineralocorticoid receptors, thereby reducing inflammation, oxidative stress, and fibrosis in the heart and kidneys [51]. The combination of SGLT2i and GLP-1RA generally shows a favorable safety profile [52]. For SGLT2i and GLP-1RA combination therapy, side effects are typically limited to those already associated with each drug class, such as genitourinary infections from SGLT2i and gastrointestinal symptoms from GLP-1RA, with no evidence of synergistic toxicity from their combined use [52]. Finerenone has an improved safety profile compared to traditional steroidal MRAs [53]. However, the real-world uptake of these combination therapies remains limited, highlighting the need for targeted implementation strategies and system-level reforms to promote equitable access to these evidence-based treatments for clinically appropriate patients.

### Limitations and future directions

This meta-analysis has limitations that should be considered when interpreting the findings. The included RCTs were not designed to directly assess the efficacy of combined GLP-1RA and SGLT2i therapy; instead, the analyses relied on post hoc assessments of baseline SGLT2i use in GLP-1RA trials, which are inherently limited by reduced statistical power and potential residual confounding [54]. Additionally, the limited number of RCTs reporting renal composite outcomes (n = 2), non-fatal myocardial infarction (n = 2), and non-fatal stroke (n = 2) led to wide confidence intervals in some cases, reducing the precision of pooled estimates. This was particularly evident in the renal composite outcome analysis, where high heterogeneity in the SGLT2i monotherapy comparator group (I^2^ = 81.6%) further complicates interpretation, likely in part due to differing outcome definitions between the AMPLITUDE-O and Harmony Outcome trials. Similarly, considerable heterogeneity was observed across multiple outcomes in the observational meta-analyses, with most showing I^2^ values > 50%. This should be considered when interpreting the results of this analysis. This was largely driven by differences in study populations, as heterogeneity was often lower in the analysis of subgroups; although, heterogeneity should be interpreted cautiously when study count is low [55]. Observational studies are also subject to unmeasured confounding [56], including socioeconomic factors, healthcare access, and lifestyle variables, which may bias results. Additionally, data on finerenone combination was limited, and definitive renal endpoint data was sparse. Lastly, this study was not prospectively registered.

Future research should prioritize RCTs specifically designed to evaluate the incremental benefits of combination therapy on hard clinical endpoints. Future studies should also examine the combined use of all three drug classes to better understand their potential additive or synergistic benefits. Further investigation into non-diabetic cohorts could uncover the utility of combination therapy in a broader patient population. Additionally, exploring the reasons for the limited use of combination therapy may help identify barriers to its implementation.

## Conclusion

This meta-analysis provides compelling evidence that dual combination therapy with GLP-1RA, SGLT2i, or finerenone offers significant cardiovascular and renal benefits in patients with T2D. RCTs show consistent effects of GLP-1RAs on cardiorenal outcomes, regardless of baseline SGLT2i use, while observational studies further support enhanced reductions in MACE, all-cause mortality, other cardiovascular endpoints, and renal outcomes with dual therapy. These findings advocate for broader clinical adoption of dual therapy in select patients. Ideally, prospective trials specifically designed to evaluate combination therapy are essential to confirm the incremental benefit and guide precise treatment strategies.

## Supplementary Information

Below is the link to the electronic supplementary material.

Supplementary Material 1

## Data Availability

No datasets were generated or analysed during the current study.
