# Supplementary material for: Cardiovascular and renal outcomes of dual combination therapies with glucagon-like peptide-1 receptor agonists and sodium-glucose transport protein 2 inhibitors: a systematic review and meta-analysis
Source: Cardiovasc Diabetol. 2025 Sep 30;24:370. doi: 10.1186/s12933-025-02900-8 (PMC12487346; doi:10.1186/s12933-025-02900-8)

**Supplementary Appendix**

**Page 2**: Search Strategy

**Page 3-5**: Background Information Tables S1-S4

**Page 6-7**: Risk of Bias Tables S5-S6

**Page 8-11**: Supplemental Figure S1-S12

**Search Strategy**

Embase search 1:

1. Glucagon-like peptide-1 receptor agonists.mp. or exp glucagon like peptide 1 receptor agonist/

2. SGLT2 inhibitor.mp. or exp sodium glucose cotransporter 2 inhibitor/

3. combination.mp. or combination drug therapy/ or drug combination/

4. 1 and 2 and 3

5. limit 4 to (human and English language)

Embase search 2:

1. exp sodium glucose cotransporter 2 inhibitor/ or sglt2.mp.

2. glp-1ra.mp. or exp glucagon like peptide 1 receptor agonist/

3. finerenone.mp. or exp finerenone/

4. 1 or 2

5. 3 and 4

6. exp drug combination/ or exp combination drug therapy/ or combination.mp.

7. combined.mp.

8. 6 or 7

9. 5 and 8

10. limit 9 to (human and English language)

MEDLINE search 1:

1. SGLT2 inhibitor.mp. or exp Sodium-Glucose Transporter 2 Inhibitors/

2. glucagon like peptide 1 receptor agonist.mp. or exp Glucagon-Like Peptide-1 Receptor Agonists/

3. 1 and 2

4. limit 3 to (english language and humans)

MEDLINE search 2:
1. exp Sodium-Glucose Transporter 2 Inhibitors/ or sglt2.mp.

2. glp-1ra.mp. or exp Glucagon-Like Peptide-1 Receptor Agonists/

3. finerenone.mp.

4. 1 or 2

5. 3 and 4

6. combination.mp. or exp Drug Therapy, Combination/

7. exp Combined Modality Therapy/ or combined.mp.

8. 6 or 7

9. 5 and 8

10. limit 9 to (english language and humans)

**Background Information Tables**

Supplementary Table S1. Baseline Characteristics of Included Randomized Controlled Trials

|  |  | **N Participants** | | **Age** ± **SD, (IQR), Years** | | **% Female Participants** | |
| --- | --- | --- | --- | --- | --- | --- | --- |
| **Trial Name**  **Study Design)** | **Population (follow up duration)** | **No SGLT2i** | **SGLT2i** | **No SGLT2i** | **SGLT2i** | **No SGLT2i** | **SGLT2i** |
| AMPLITUDE-O (RCT) | T2D with CVD or CKD (median: 1.81 years) | 3458 | 618 | 64.6 ± 8.3 | 64.0 ± 8.1 | 33.8% | 28.2% |
| FLOW (RCT) | T2D and CKD (median: 3.4 years) | 2983 | 550 | 67 ± 8.9 | 64.8 ± 9.1 | 31.7% | 22.5% |
| Harmony Outcomes (RCT) | T2D and CVD (median: 1.6 years) | 8887 | 575 | 64.2 ± 8.7 | 62.9 ± 8.2 | 31% | 24.3% |
| SOUL (RCT) | T2D with ASCVD and/or CKD (mean: 47.5 months) | 7054 | 2596 | 67.0 (IQR 61.0–72.0) | 65.0 (IQR 59.0–70.0) | 31.4% | 22.2% |

Supplementary Table S2. Baseline Characteristics of Included Observational Studies

| **Author**  **Study Design)** | **Population (follow up duration)** | **N Participants** | | | **Age** ± **SD, [IQR], Years** | | | **% Female Participants** | | |
| --- | --- | --- | --- | --- | --- | --- | --- | --- | --- | --- |
|  |  | **SGLT2i Monotherapy** | **GLP1-RA Monotherapy** | **Combination Therapy** | **SGLT2i Monotherapy** | **GLP1-RA Monotherapy** | **Combination Therapy** | **SGLT2i Monotherapy** | **GLP1-RA Monotherapy** | **Combination Therapy** |
| Lopez et al. 2022 (Retrospective cohort) | T2D with ASCVD and HFrEF (Median: 842 days) | 343 | NR | 343 | 68.0 ± 7.8 | NR | 67.7 ± 7.5 | 0.9% | NR | 0.9% |
| Riley et al. 2023 (Retrospective cohort) | T2D on insulin (up to five years after index date) | 143740 | 186844 | 108507 | 62.8 ± 12.2 | 58.7 ± 13.0 | 58.7 ± 11.5 | 40.3% | 56.4% | 49.0% |
| Garcia-Vega et al. 2024 (non- concurrent prospective cohort) | Majority T2D*  (Median: 19 months) | 12029 | 1071 | 2449 | 70 (62-77) | 67 (58-75) | 65 (57-72) | 40% | 53% | 43% |
| Jhu et al. 2024 (Retrospective cohort) | T2D (up to five years after index date) | 71186 | NR | 71186 | 57.2 ± 11.7 | NR | 57.1 ± 10.8 | 45% | NR | 45.6% |
| Marfella et al. 2024 (Prospective cohort) | T2D and MI (24 month) | 99 | 130 | 214 | 68 (63–73) | 67 (61–70) | 70 (64–75) | 35.4% | 40% | 38.8% |
| Patel et al. 2024 (Retrospective Cohort) | T2D with BMI>27 and HFpEF (12 months) | 7044 | NR | 7044 | NR | 63.4 ±11.8 | 63.2 ± 10.5 | 43.9% | NR | 43.7% |
| Simms-Williams et al. 2024 (Retrospective Cohort) | T2D (Between 8.4-9.1 months) | 8942 | 6696 | 6696 | 57.5 (10.3) | 57.3 (10.4) | 56.7 (10.4) | 46.9 | 45.6 | 45.5 |
| Chaiyakunapruk et al. 2025 (Retrospective Cohort) | T2D with ASCVD (Minimum of >6 months follow up within ~5.5 year observation window) | 130220 | NR | 34690 | 63.7 (10.5) | NR | 63.7 (10.5) | 35.9% | NR | 39.3% |
| Liu et al. 2025 (Retrospective Cohort) | T2D with ACS (1 year) | 208 | NR | 208 | 62.7 ± 11.7 | NR | 63.2 ± 10.9 | 34.1% | NR | 30.3% |

NR = Not relevant, * 97% of patients had type 2 diabetes

Supplementary Table S3. Baseline Characteristics of Included Observational Studies

| **Trial Name**  **Study Design)** | **Population (follow up duration)** | **N Participants** | | **Age (SD) Years** | | **% Female Participants** | | **% Type 2 Diabetes** | |
| --- | --- | --- | --- | --- | --- | --- | --- | --- | --- |
|  |  | **Finerenone Monotherapy** | **Combination Therapy** | **Finerenone Monotherapy** | **Combination Therapy** | **Finerenone Monotherapy** | **Combination Therapy** | **Finerenone Monotherapy** | **Combination Therapy** |
| Chuang et al. 2025 (Retrospective Cohort) | CKD patients, majority of which had T2D* (Combined vs finerenone: 13.0 ± 9.9 months Combined vs SGLT-2 inhibitor: 12.2 ± 9.7 months) | 643 | 643 | 67.5 ± 11.5 | 67.0 ± 11.2 | 39.5% | 39.3% | 87.4% | 87.1% |
|  |  | **SGLT2i Monotherapy** | **Combination Therapy** | **SGLT2i Monotherapy** | **Combination Therapy** | **SGLT2i Monotherapy** | **Combination Therapy** | **SGLT2i Monotherapy** | **Combination Therapy** |
|  |  | 850 | 850 | 66.6 ± 12.1 | 66.7 ± 11.4 | 35.6% | 35.1% | 88.1% | 89.2% |

* Prevalence of T2D ranged from 87.1% to 89.2%

Supplementary Table S4. Population of Included Observational Studies

| Author | Population |
| --- | --- |
| Lopez et al. 2022 | Patients all had T2D, established ASCVD (ischemic heart disease, cerebrovascular disease, or peripheral artery disease), and HFrEF documented as left-ventricular ejection fraction ≤ 40 %. |
| Riley et al. 2023 | Patients with T2D receiving insulin. |
| Garcia-Vega et al. 2024 | Inclusion criteria was limited to the prescription of new SGLT2i or GLP-1RA within a pre-defined time range, although the population had a high prevalence of T2D (97% of included patients). |
| Jhu et al. 2024 | T2D with eGFR ≥60 mL/min/1.73 m2 |
| Marfella et al. 2024 | Patient with T2D, hospitalized for first acute MI, treated for at least 3 months prior to hospitalization. |
| Patel et al. 2024 | Patients with T2D, BMI ≥ 27 kg/m², and HFpEF (defined as LVEF ≥ 45%). |
| Simms-Williams et al. 2024 | Patients with T2D. |
| Chaiyakunapruk et al. 2025 | Patients with T2D and documented history of ASCVD. |
| Liu et al. 2025 | Patients with T2D who had been taking an SGLT-2 inhibitor for >3 months before their acute coronary syndrome admission. |
| Chuang et al. 2025 | Patients with CKD (diagnosis code for CKD with at least one of: eGFR <60 mL/min/1.73 m2, diagnosis of proteinuria, urine albumin/creatinine ratio ≥30 mg/g, or urine protein/creatinine ratio ≥150 mg/g. Although not part of formal inclusion criteria, the population had a high prevalence of T2D (87.1% to 89.2%). |

**Risk of Bias Tables**

Supplementary Table S5 - Risk of Bias 2.0 Outcomes for Randomized Control Trial

|  | Randomization process | Intervention deviation | Missing outcome data | Measurement of outcomes | Reporting of the outcome | Overall |
| --- | --- | --- | --- | --- | --- | --- |
| Hernandez et al. 2018 (Harmony Outcomes) | Low | Low | Low | Low | Low | Low |
| Gerstein et al. 2021 (AMPLITUDE-O) | Low | Low | Low | Low | Low | Low |
| Perkovic et al. 2024 (FLOW) | Low | Low | Low | Low | Low | Low |
| McGuire et al. 2025 (SOUL) | Low | Low | Low | Low | Low | Low |

Supplementary Table S6 - Newcastle-Ottawa Scale Outcomes for Observational Trials

| Authors (Year) | Study Type | Selection | | | | Comparability | Outcome | | | Final Score |
| --- | --- | --- | --- | --- | --- | --- | --- | --- | --- | --- |
|  |  | Representativeness of the exposed cohort | Selection of  the  non-exposed  cohort | Ascertainment  of exposure | Demonstration that  outcome of interest was  not present at start of  study | Comparability of  cohorts on the basis  of the design or  Analysis | Assessment  of outcome | Was follow-up  long enough for  outcomes to  occur | Adequacy  of follow  up of  cohorts |  |
| Lopez et al. 2022 | Retrospective cohort | * | * | * |  | ** | * | * |  | 7/9 |
| Riley et al. 2023 | Retrospective cohort | * | * | * | * | ** | * | * |  | 8/9 |
| Garcia-Vega et al. 2024 | Non- concurrent prospective cohort | * | * | * | * | ** | * | * | * | 9/9 |
| Jhu et al. 2024 | Retrospective cohort | * | * | * | * | ** | * | * |  | 8/9 |
| Marfella et al. 2024 | Prospective cohort | * | * | * | * | ** | * | * | * | 9/9 |
| Patel et al. 2024 | Retrospective cohort | * | * | * |  | ** | * | * | * | 8/9 |
| Simms-Williams et al. 2024 | Retrospective cohort | * | * | * |  | ** | * | * | * | 8/9 |
| Chaiyakunapruk et al. 2025 | Retrospective cohort | * | * | * | * | ** | * | * | * | 9/9 |
| Liu et al. 2025 | Retrospective cohort | * | * | * | * | ** | * | * |  | 8/9 |
| Chuang et al. 2025 | Retrospective cohort | * | * | * | * | ** | * | * | * | 9/9 |

**Supplemental Figures**

Figure S1 - Study selection process


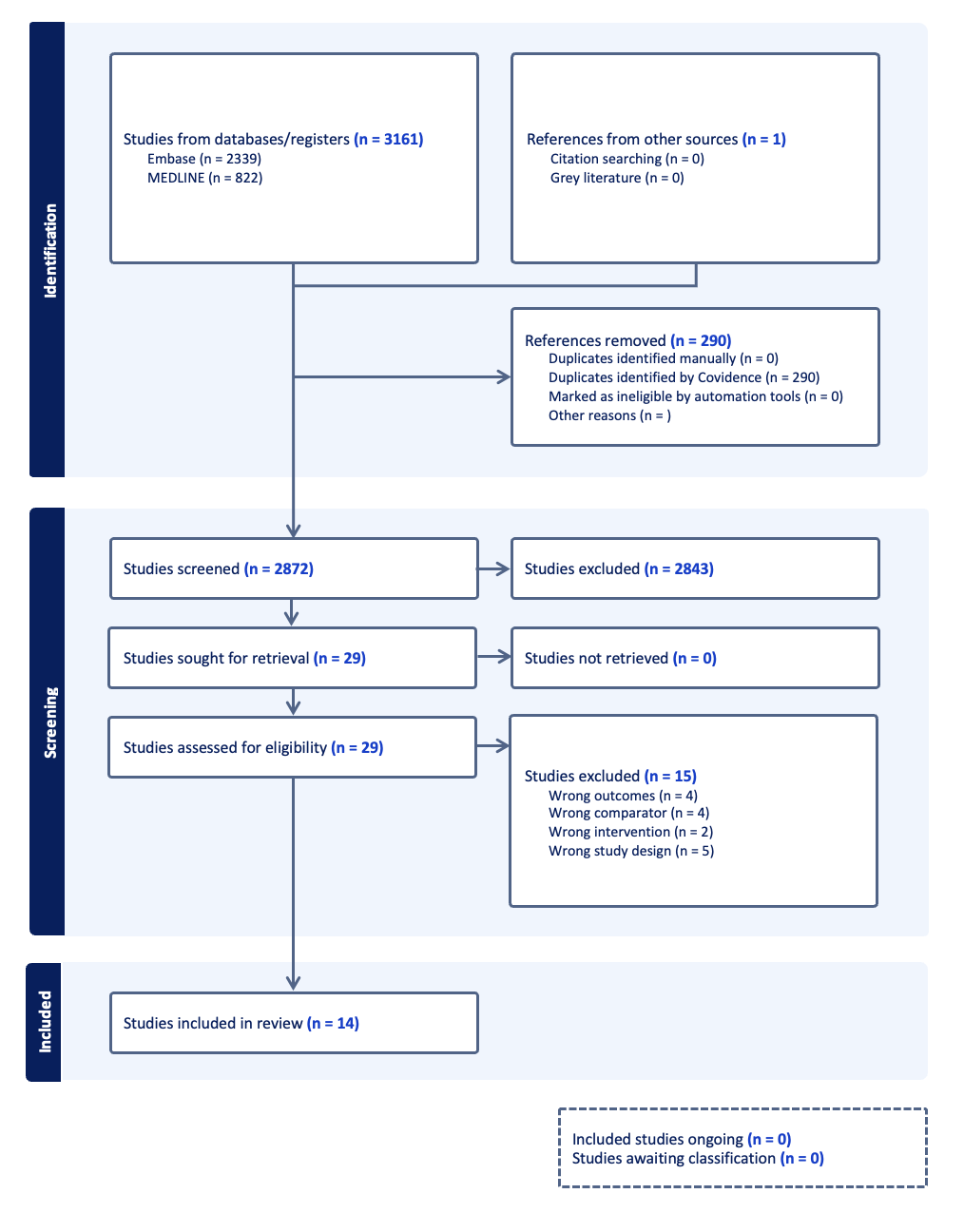


**Subgroup Analysis Data for Observational Studies**

Figure S2 - MACE (Broader T2D population, excluding studies restricted to T2D with major comorbidities such as ASCVD, MI, HF, or those limited to insulin-treated T2D)


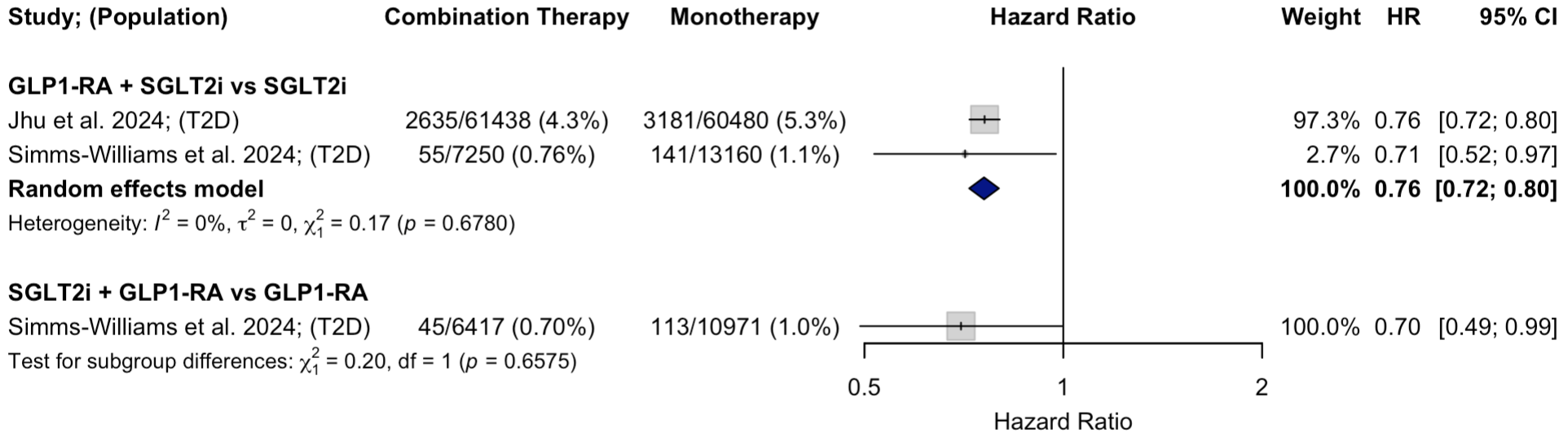


Figure S3 - MACE (studies in T2D populations with comorbid MI, ACS, or established ASCVD)


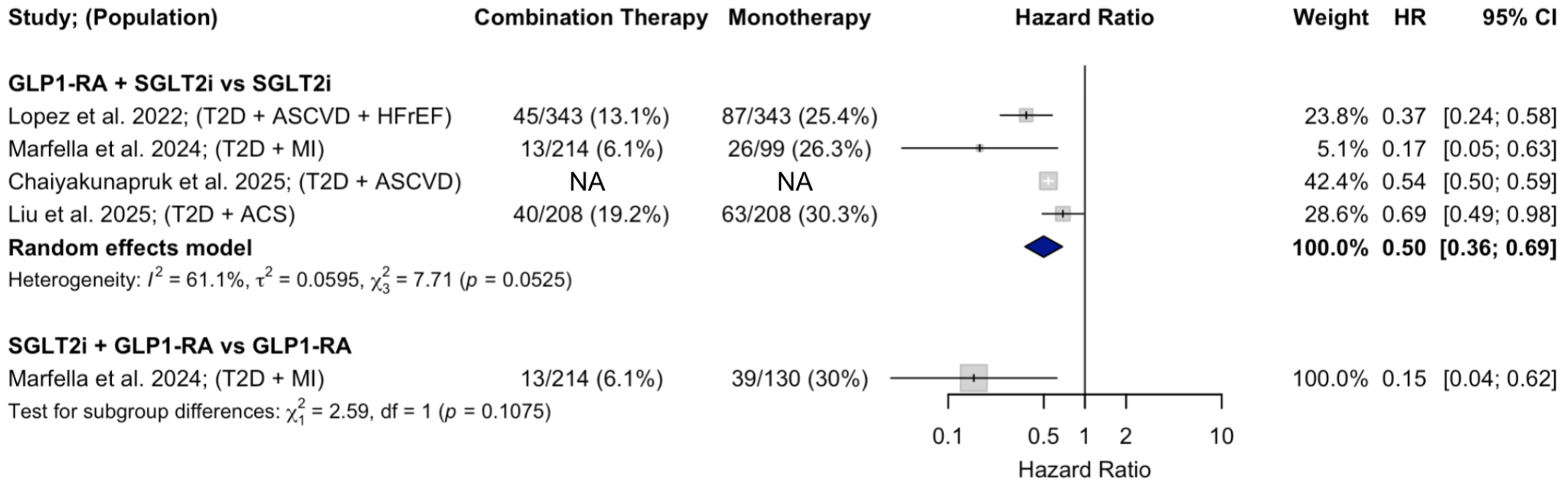


Figure S4 - CV Mortality (Broader T2D population, excluding studies restricted to T2D with major comorbidities such as ASCVD, MI, HF, or those limited to insulin-treated T2D)


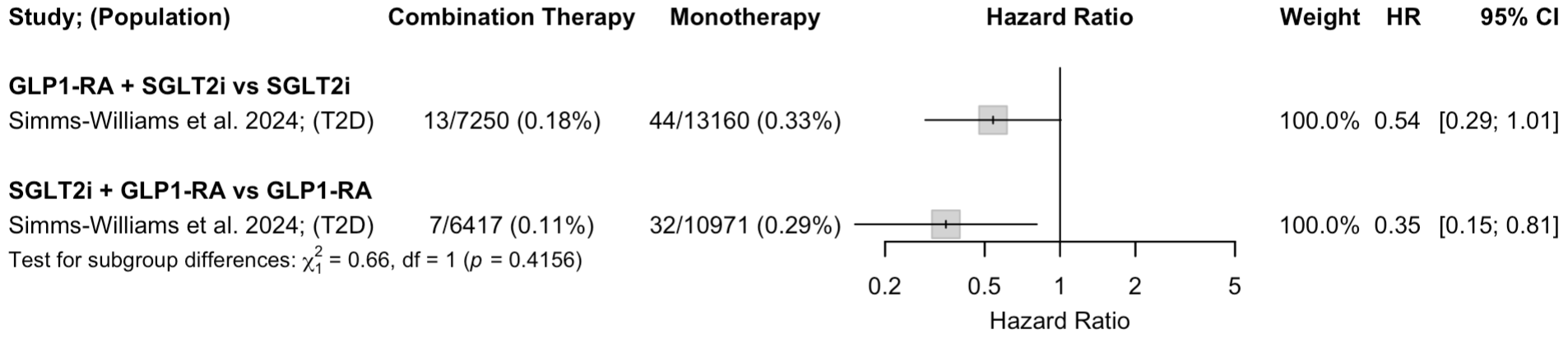


Figure S5 - CV Mortality (studies in T2D populations with comorbid MI, ACS, or established ASCVD)


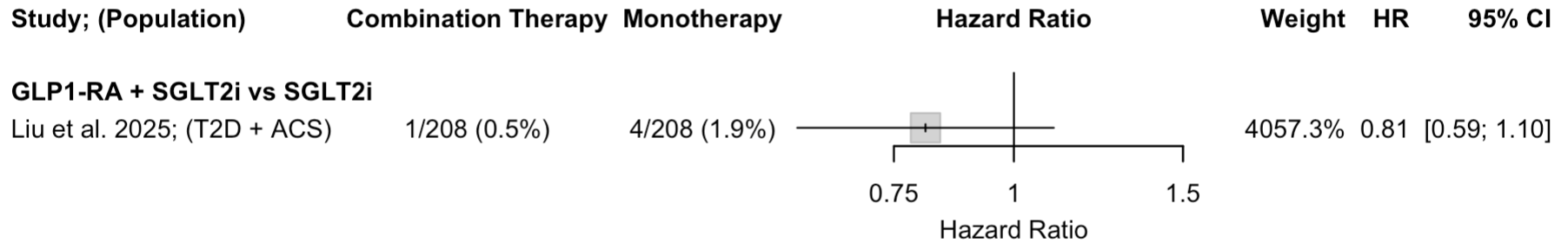


Figure S6 - Myocardial Infarction (Broader T2D population, excluding studies restricted to T2D with major comorbidities such as ASCVD, MI, HF, or those limited to insulin-treated T2D)


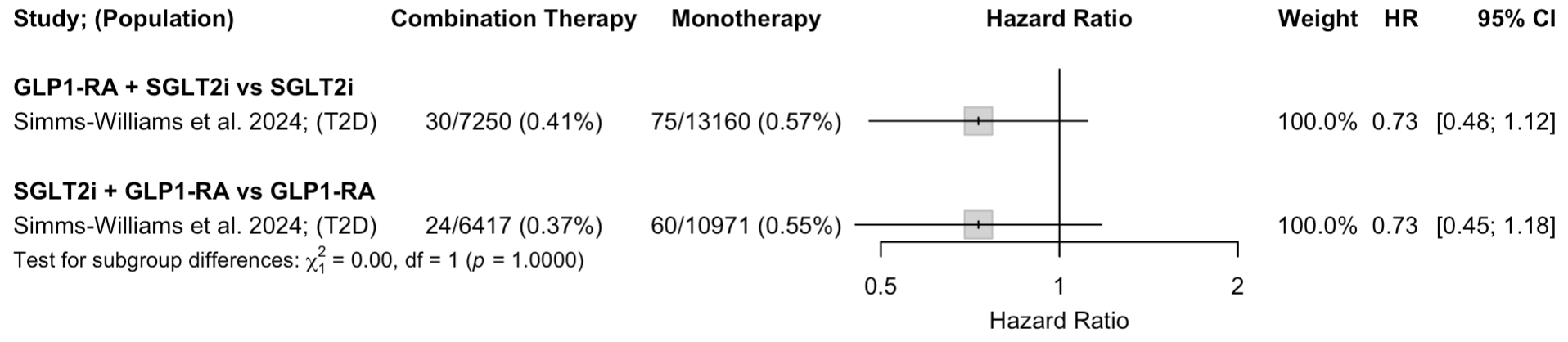


Figure S7 - Myocardial Infarction (studies in T2D populations with comorbid MI, ACS, or established ASCVD)


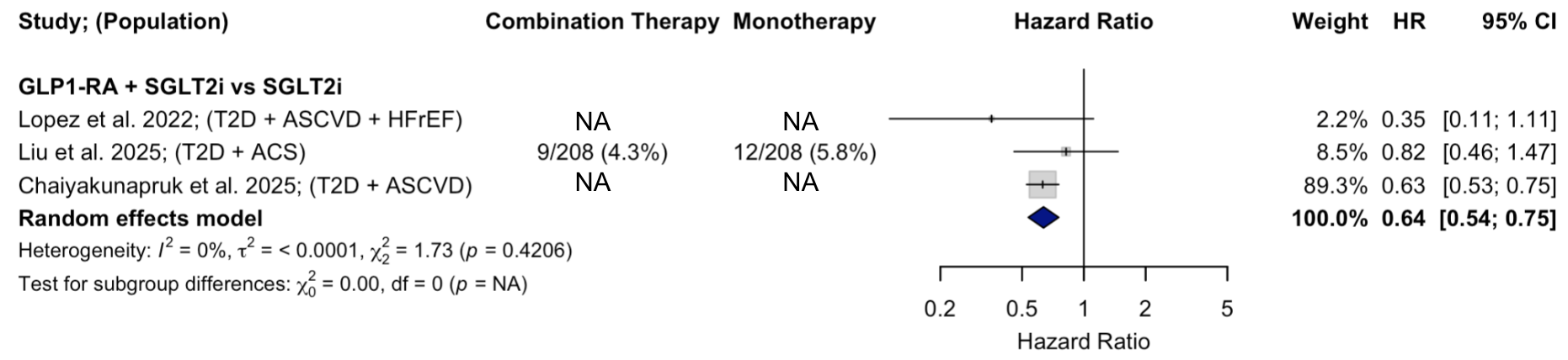


Figure S8 - Stroke (Broader T2D population, excluding studies restricted to T2D with major comorbidities such as ASCVD, MI, HF, or those limited to insulin-treated T2D)


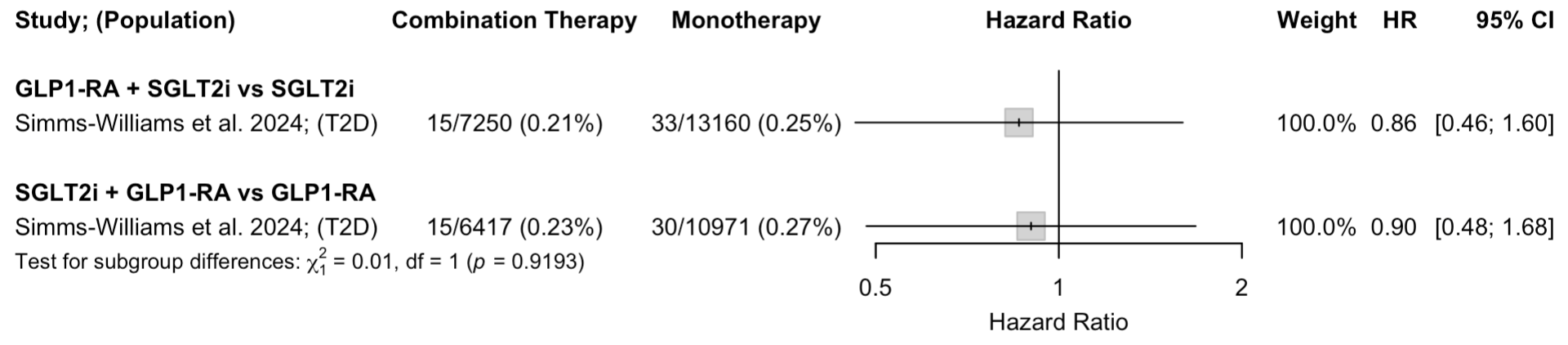


Figure S9 - Stroke (studies in T2D populations with comorbid MI, ACS, or established ASCVD)


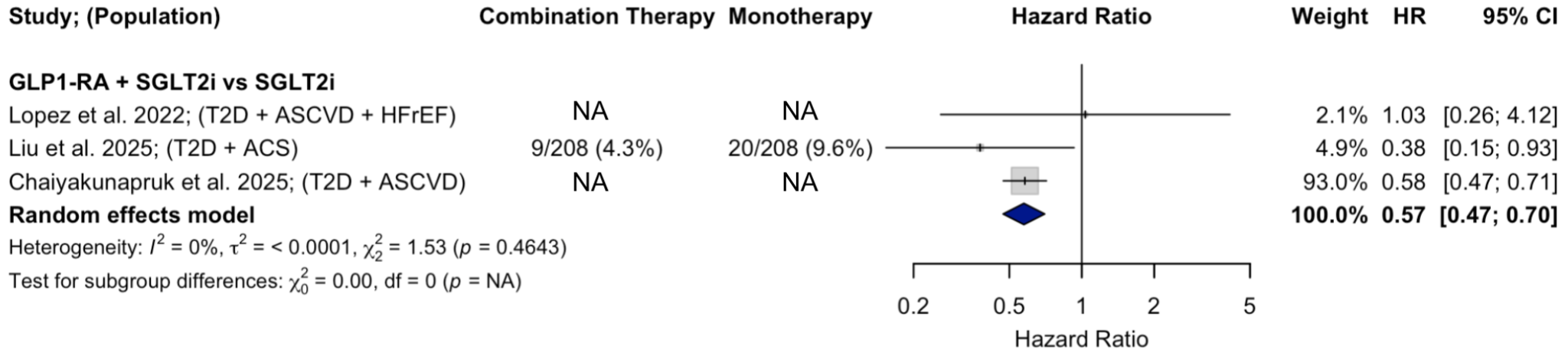


Figure S10 - All-Cause Mortality (Broader T2D population, excluding studies restricted to T2D with major comorbidities such as ASCVD, MI, HF, or those limited to insulin-treated T2D)


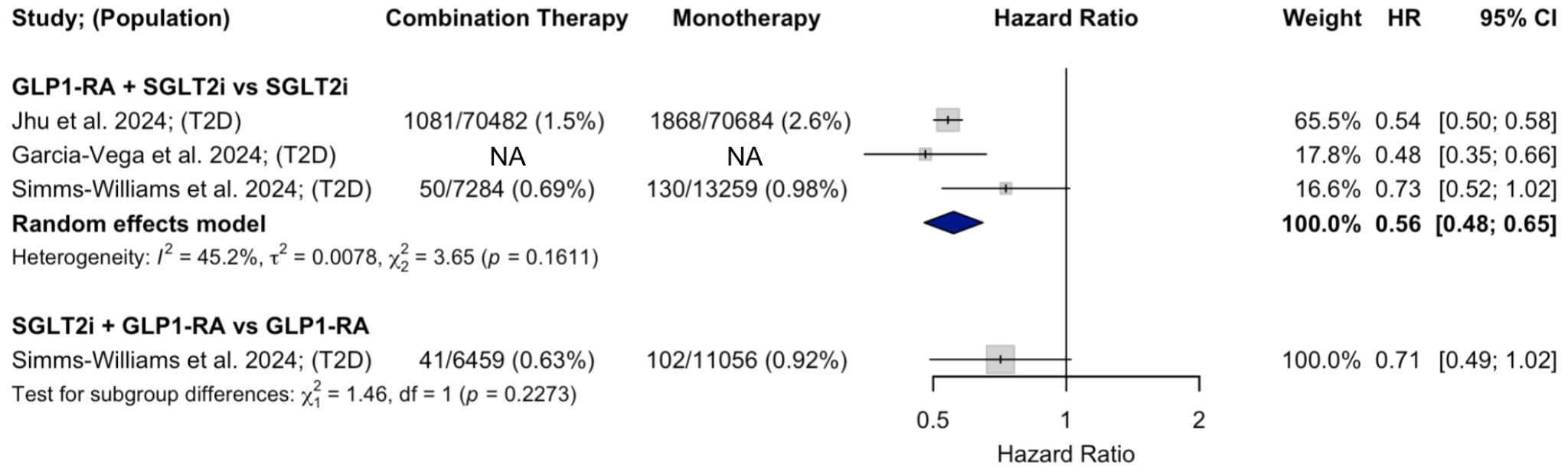


Figure S11 - All-Cause Mortality (studies in T2D populations with comorbid MI, ACS, or established ASCVD)


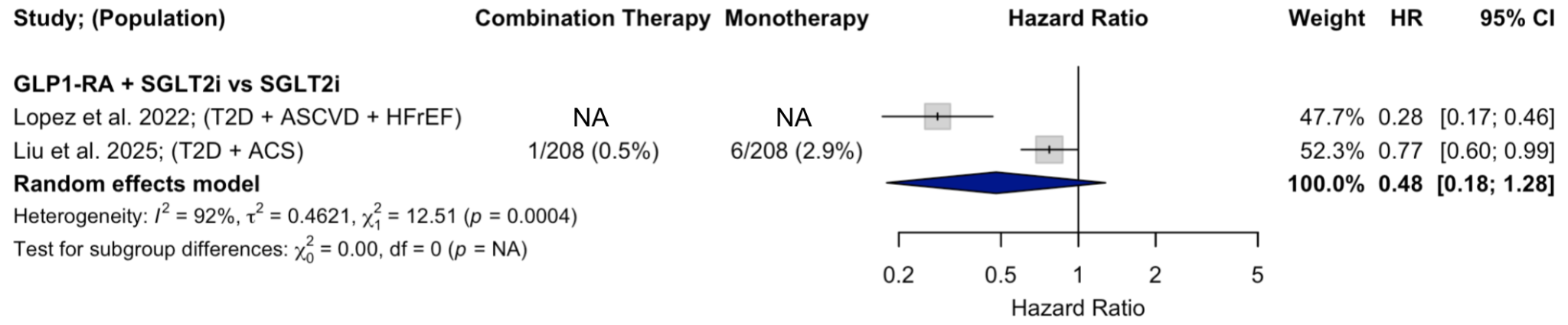


Figure S12 - Heart Failure Hospitalization (Broader T2D population, excluding studies restricted to T2D with major comorbidities such as ASCVD, MI, HF, or those limited to insulin-treated T2D)


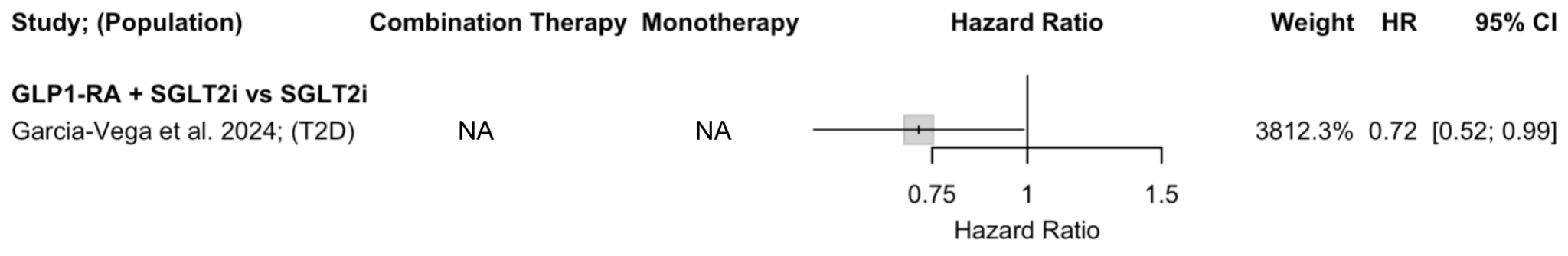

Supplement: Supplementary file 1 — Supplementary Material 1 [file 12933_2025_2900_MOESM1_ESM.docx]
